# Actions of Metformin in the Brain: A New Perspective of Metformin Treatments in Related Neurological Disorders

**DOI:** 10.3390/ijms23158281

**Published:** 2022-07-27

**Authors:** Nuojin Li, Tian Zhou, Erkang Fei

**Affiliations:** 1 Queen Mary School of Nanchang University, Nanchang 330031, China; 4217118053@email.ncu.edu.cn; 2 School of Basic Medical Sciences, Nanchang University, Nanchang 330031, China; zhoutian@ncu.edu.cn; 3 Institute of Life Science, Nanchang University, Nanchang 330031, China

**Keywords:** metformin, synapse, synaptic transmission, neurological disorders

## Abstract

Metformin is a first-line drug for treating type 2 diabetes mellitus (T2DM) and one of the most commonly prescribed drugs in the world. Besides its hypoglycemic effects, metformin also can improve cognitive or mood functions in some T2DM patients; moreover, it has been reported that metformin exerts beneficial effects on many neurological disorders, including major depressive disorder (MDD), Alzheimer’s disease (AD) and Fragile X syndrome (FXS); however, the mechanism underlying metformin in the brain is not fully understood. Neurotransmission between neurons is fundamental for brain functions, and its defects have been implicated in many neurological disorders. Recent studies suggest that metformin appears not only to regulate synaptic transmission or plasticity in pathological conditions but also to regulate the balance of excitation and inhibition (E/I balance) in neural networks. In this review, we focused on and reviewed the roles of metformin in brain functions and related neurological disorders, which would give us a deeper understanding of the actions of metformin in the brain.

## 1. Introduction

Metformin is a first-line drug for type 2 diabetes mellitus (T2DM) therapy with a long history. As a biguanide derivative, metformin was isolated from the extracts of the plant French lilac (*Galega officinalis*) in the 1920s [1]. Then, metformin was approved for diabetes mellitus treatment in Europe and Canada in 1957 and in the USA in 1995 [2]. Metformin has been prescribed for more than 60 years, characterized by good safety, high efficiency of blood glucose control, and clear but not fully understood cardioprotective effect. The alteration in cellular energy metabolism is the core mechanism of metformin’s action [3]. The best-known hypoglycemic effects of metformin are achieved by multiple mechanisms: inhibition of liver gluconeogenesis and intestinal glucose uptake, an increase of glucose uptake in peripheral tissues, and improvement of peripheral insulin sensitivity [4]. 

T2DM leads to brain structural and functional changes and increases the risk of neurological disorders’ comorbidities. Alzheimer’s disease (AD), the most common neurodegenerative disorder, has been shown by many studies to be closely associated with T2DM [5,6]. Compared with healthy individuals, people with T2DM have a significantly increased risk of developing AD [7,8]. T2DM is also linked to another common neurodegenerative disorder, Parkinson’s disease (PD) [9,10]. Epidemiological studies have shown that patients with T2DM have a higher risk of PD and faster progression of PD symptoms [11,12]. In addition to neurodegenerative disorders, T2DM is associated with psychiatric disorders, too. The prevalence of schizophrenia and major depressive disorder (MDD) in patients with T2DM is higher than that in the general population [13,14]. In a large-scale meta-analysis, people with schizophrenia, bipolar disorder and MDD have a higher risk of developing T2DM than healthy controls [15].

Neurological disorders are the second leading cause of death worldwide. Current therapeutic strategies have not been very successful in treating these disorders: a combination of most central nervous system (CNS) drugs that target symptoms rather than etiology, a lack of safe and effective drugs, and difficulties in clinical drug development [16]. And diabetes drugs, including metformin, may be potential drugs for treating neurological disorders, providing new ideas for their treatments [17,18]. Finding new uses for old drugs is a current hot spot, and metformin has been reported in clinical and animal studies to exert neuroprotective effects in many neurological disorders [19,20,21,22].

Neurons are the principal cells in the brain. Communications between neurons via synapses are fundamental for brain functions, and their defects have been implicated in many neurological disorders. Abnormal synaptic transmission, neuronal dysregulation and neuroinflammation in the brain are common pathological manifestations of neurological disorders. In this review, we reviewed the studies of metformin in neurological disorders and focused on the mechanisms underlying the actions of metformin in the brain, which would provide new insight into the treatments of neurological disorders.

## 2. General Mechanism Underlying the Hypoglycemic Effects of Metformin

**Figure 1:**
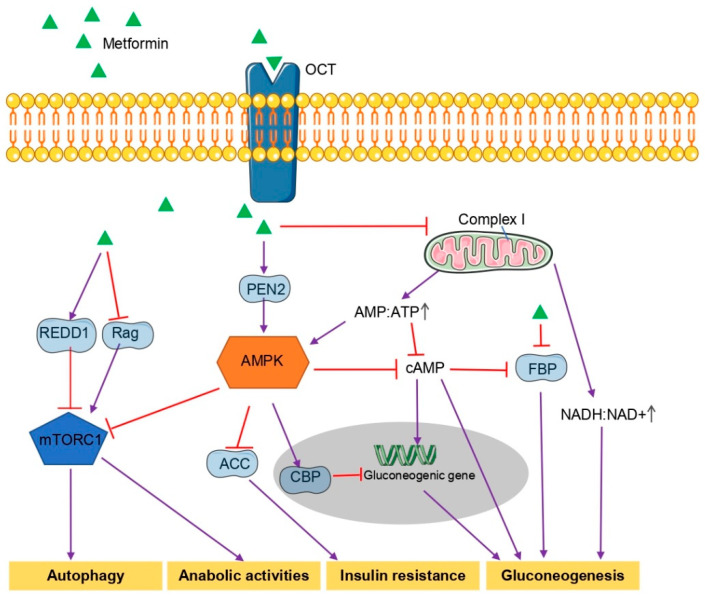
The general mechanism underlying the hypoglycemic effects of metformin. Metformin activates AMPK through lysosomal or mitochondrial mechanisms. AMPK increases insulin sensitivity by inhibiting ACC, activates CBP to inhibit gluconeogenesis gene expression, inhibits mTORC1 to suppress cellular anabolic activity, and inhibits cAMP production to reduce gluconeogenesis. In addition, metformin can also achieve glucose reduction in a non-AMPK-dependent manner by inhibiting AMP:ATP ratio and NADH:NAD+ ratio through mitochondrial mechanisms or by directly targeting FBP. REDD1, regulated in development and DNA damage response 1; Rag, Rag family of GTPases; PEN2, presenilin enhancer 2; AMPK, 5’-AMP-activated protein kinase; mTORC1, mechanistic target of rapamycin complex 1; ACC, acetyl CoA carboxylase; FBP, fructose-1,6-bisphosphatase; CBP, CREB binding protein; OCT, organic cation transporters; ATP, Adenosine triphosphate; AMP, adenosine monophosphate; NADH, the reduced form of nicotinamide adenine dinucleotide; NAD+, the oxidized form of nicotinamide adenine dinucleotide.

Nowadays, metformin is widely recognized as a 5’-AMP-activated protein kinase (AMPK) agonist (Figure 1). Metformin induces energy stress by inhibiting the mitochondrial respiratory chain complex I, decreasing ATP production, increasing AMP and ADP production, and thus increasing AMP/ATP ratio [23]. Next, glucagon-induced cyclic AMP (cAMP) synthesis is inhibited, and AMPK is activated [24]. AMPK is able to sense low ATP levels through v-ATPase, switch cells from an anabolic to a catabolic state, promote mitochondrial biogenesis and regulate autophagy [25,26]. Recently, a study found presenilin enhancer 2 (PEN2) is a new target of metformin [26]. At a low concentration of metformin, PEN2 binds to and inhibits v-ATPase activity and finally activates lysosomal AMPK without increasing AMP [26]. Metformin-mediated AMPK activation leads to the decrease of acetyl CoA carboxylase (ACC) activity, induces fatty acid oxidation and inhibits the expression of lipogenic enzymes [27]. In an AMPK-dependent pathway, metformin promotes small heterodimer partner (SHP) protein production and ameliorates hepatic insulin resistance by regulating gluconeogenesis and insulin sensitivity [28]. Metformin inhibits the expression of gluconeogenic genes by stimulating the phosphorylation of cAMP-response element binding protein (CREB) binding protein [29]. Furthermore, metformin-induced AMPK activation inhibits the mechanistic target of rapamycin (mTOR) complex 1 (mTORC1) signaling via directly suppressing Raptor, a key component of mTORC1 [2] or indirectly activating the tuberous sclerosis complex [30,31]; moreover, in rat models of obesity and diabetes, metformin activates the duodenal AMPK-dependent pathway to reduce liver gluconeogenesis and blood glucose levels [32].

However, in genetic loss-of-function experiments, like liver AMPK-deficient mice, metformin still lowers blood glucose levels, suggesting AMPK-independent gluconeogenesis [33]. In a non-AMPK-dependent manner, metformin antagonized the hyperglycemic effect of hepatic glucagon signal by reducing cAMP production [24]; moreover, metformin can inhibit mitochondrial glycerophosphate dehydrogenase, leading to an altered hepatocellular redox state and reduced hepatic gluconeogenesis [34]. Recently, fructose-1,6-bisphosphatase-1 (FBP1), a rate-controlling enzyme in gluconeogenesis, has been confirmed to be a direct target of metformin [35]. Metformin, independent of AMPK, inhibits mTORC1 by inhibiting the Rag family of GTPases [36] or upregulating regulated in development and DNA damage response 1 (REDD1, a negative regulator of mTORC1) [37]. In in vivo experiments in rats, metformin inhibited gluconeogenesis of lactate and glycerol independently of complex I inhibition and was associated with an increase in the cellular redox state [38]. 

## 3. Metformin in Neurological Disorders

### 3.1. Alzheimer’s Disease (AD)

AD, the most common form of dementia, is characterized by amyloid plaques and neurofibrillary tangles in the brain, with loss of synapses and neurons, leading to cognitive dysfunction and ultimately dementia [39]. T2DM is a significant risk factor for the development of dementia, including AD. Many clinical randomized controlled trials have found that metformin monotherapy or combination therapy with sulfonylurea can significantly reduce the risk of dementia in T2DM patients [40,41,42,43]. Two meta-analyses supported the above conclusions [44,45]. In an observational cohort study of 145,928 older T2DM patients, metformin users only reduced the dementia risk by 4 percent, compared with 47 percent for pioglitazone [46]. Furthermore, some studies found that metformin improved cognitive impairment in diabetes patients [47] and executive function in AD patients [48]. Contrary to the above findings, several investigations have proved that metformin increased the risk of AD [49,50] and was associated with worse cognitive performance [51]. Differences in metformin’s effect on cognitive function may result from individual differences in genetic composition. In *APOE* ε4 carriers of AD, metformin use was associated with a faster rate of delayed memory decline [52]. Taken together, most articles drew a conclusion that metformin can reduce the risk of dementia or AD in T2DM patients.

The effect of metformin on cognitive function in preclinical studies is also controversial. Metformin can reduce tau phosphorylation and total tau levels, but its effect on amyloid-beta (Aβ) production remains inconsistent. In amyloid-beta precursor protein/presenilin 1 (APP/PS1, an AD mouse model) mice, metformin attenuated spatial memory deficit, neuronal loss, increased Aβ plaque and chronic inflammation [53]. Metformin improved memory in the senescence-accelerated mouse prone 8 (SAMP8) mouse model of spontaneous onset AD by decreasing APPc99 and p-tau [54]. In a protein phosphatase 2A (PP2A)-dependent way, metformin reduces tau phosphorylation in a cellular model [55]. Metformin increased the production of Aβ peptides by upregulating β-site amyloid precursor protein cleaving enzyme-1 (BACE1) activity in an AMPK-dependent manner, which may explain the mechanism of adverse results of metformin in AD [56]. Although metformin enhances Aβ production in vitro, in vivo studies revealed metformin could alleviate the deposition of Aβ; a different experimental system may contribute to the opposite results. 

### 3.2. Parkinson’s Disease (PD)

PD is a neurodegenerative disease, the hallmark of which is the death of dopaminergic neurons in substantia nigra pars compacta, post-translational modification and agglomeration of α-synuclein, mitochondrial dysfunction, and oxidative stress [57]. There is growing evidence that T2DM patients have a higher risk of PD and share similar dysfunctional pathways, suggesting a common underlying pathological mechanism [58]. In a clinical study, compared with no oral anti-hyperglycemic agents, metformin alone did not affect the risk of PD; however, metformin combined with sulfonylureas reduced the incidence of PD compared with sulfonylureas alone [59]. Compared with the glitazone user, metformin monotherapy was associated with a significantly higher incidence of PD [60]. In a cohort study, the metformin cohort exhibited a higher risk of PD than the non-metformin cohort [49]. In sum, most clinical studies suggested that metformin has no effect or even negative effect on PD.

However, metformin has shown good neuroprotective effects in PD animal models. In 1-Methyl-4-phenyl-1,2,3,6-tetrahydropyridine (MPTP)-induced PD mice, long-term metformin treatment significantly improved locomotor and muscular activities [61]. In the branched-chain amino acid transferase 1 (bcat-1) knockdown worm model of PD, metformin treatment could correct the abnormal mitochondrial respiration and evidently rescued dopamine neuron viability [62]. In the 6-hydroxydopamine (6-OHDA)-lesioned mouse model of PD, metformin suppresses the development of dyskinesia and regulates Akt and glycogen synthase kinase 3 (GSK3) signaling and astrocyte activation [63,64]. In the lipopolysaccharide (LPS)-induced rat model of PD, metformin generally inhibited the activation of microglia and the expression of inflammatory cytokines [65]. In the haloperidol-induced catalepsy model of PD, metformin significantly attenuated memory deficit, oxidative stress and lipid peroxidation [66].

### 3.3. Huntington’s Disease (HD)

HD is a progressive neurodegenerative disease characterized by expanded CAG repeat in the gene encoding huntingtin, resulting in abnormally long polyglutamine (polyQ) repeat in the huntingtin protein [67]. HD patients with T2DM receiving metformin had better cognitive test results than those without diabetes not taking metformin [68]. In Hdh150 knock-in mice of the premanifest HD model, metformin can reduce the aberrant huntingtin load and completely restore the early network activity pattern and abnormal behavior [21]. In two other HD animal models, metformin improved motor and neuropsychiatric phenotypes in zQ175 mice, reduced polyglutamine aggregation, and restored neuronal function through mechanisms via AMPK in worm models of polyglutamine toxicity [69,70]. 

### 3.4. Major Depressive Disorder (MDD)

MDD is a heterogeneous disorder whose pathophysiology is not fully understood, and effective biomarkers are lacking [71]. In a large-scale metformin study of adolescents with severe mental illness (schizophrenia spectrum disorder, bipolar spectrum disorder or psychotic depression), metformin add-on was associated with significantly fewer reports of aggressive/hostile and impulsive problems than the control group [22]. The addition of metformin had no significant difference compared to the control group, but both alleviated the weight gain caused by antipsychotics in children and had no undesirable adverse effects [22]. In a nationwide population-based study, continued use of metformin and combination therapy is associated with a lower incidence rate of depression, while pioglitazone was not [72]. In a double-blind placebo-controlled trial, MDD patients who take metformin as an adjunct to fluoxetine have a better Hamilton Depression Rating Scale (HDRS) score, and the response and remission rates have been increased compared to the placebo group [73]. In a clinical study, all subjects were post-stroke depression combined with T2DM and took fluoxetine [74]. Compared with before taking the drug, the metformin subgroup had a slight improvement in the symptoms of depression, but pioglitazone had a more significant antidepressant effect [74]. In a six-week double-blind study of 50 patients with polycystic ovary syndrome (PCOS) and MDD, pioglitazone was superior to metformin in reducing HDRS scores at the end of the study [75]. In a randomized controlled trial of overweight adults, metformin showed a slight but statistically significant improvement in the Quality of Well-being Scale and the Beck Depression Inventory (BDI) [76]. In a clinical study, chronic metformin treatment significantly improved cognitive function in female diabetic or prediabetic patients with MDD [77]. After the 12-week metformin intervention, PCOS subjects slightly improved their BDI score [78]. Taken together, most clinical studies of depression-related disorders support that metformin is beneficial for alleviating depressive symptoms.

In animal experiments, metformin alleviated depressive-like symptoms caused by external stimuli. In the chronic social defeat stress (CSDS)-induced depression mice model, metformin alleviates depression-like behavior, improves CSDS-induced synaptic defects, and upregulates brain-derived neurotrophic factor (BDNF) expression by activating AMPK/CREB signaling pathways [79]. In LPS-treated mice, metformin administration ameliorated depressive-like behaviors and corrected abnormal glutamatergic transmission [80]. Metformin in high-fat diet (HFD) induced insulin-resistant mice stimulated 5-hydroxytryptamine (5-HT) neurons excitability and 5-HT neurotransmission while hindering HFD-induced anxiety by decreasing circulating branched-chain amino acids [81]. Metformin can also attenuate depression-like behaviors in corticosterone-induced mice with metabolic disturbance [82]; therefore, metformin has antidepressant effects. 

### 3.5. Fragile X Syndrome (FXS)

In seven FXS patients who received metformin, consistent improvements in irritability, social responsiveness, hyperactivity and social avoidance were observed [83]. Metformin in a drosophila FXS model rescued long-term memory defects and improved olfactory learning [84]. In *Fmr1* knockout (KO) mouse model of FXS, metformin reverses the increased grooming and social behavior deficits, rescues long-term depression and impaired spine morphology and selectively normalizes extracellular regulated kinase (ERK) signaling and the expression of matrix metalloproteinase-9 (MMP-9) [85]. 

## 4. Potential Mechanisms for Actions of Metformin in the Brain

### 4.1. Blood–Brain Barrier (BBB)

BBB is a selective barrier, sheathed by mural vascular cells and perivascular astrocyte end-feet and formed by continuous endothelial cells that line cerebral microvessels [86]. The destruction of the BBB is related to neuroinflammation, neuronal injury and synaptic dysfunction, which leads to various neurodegenerative pathways [87]. BBB is a complex, dynamic interface, and achieving sufficient BBB penetration is a great challenge for treating CNS diseases [88]. Oral metformin can quickly cross the BBB and accumulate in the structure of the CNS [89]. Metformin concentration in the cerebrospinal fluid reached the peak of 29 μM 30 min after metformin (200 mg/kg i.p.) was administered to C57Bl6 mice [90].

In rat brain microvascular endothelial cells, metformin induces upregulation of BBB functions by AMPK activation [91]. Metformin treatment can protect the tight junction of endothelial cells, prevent the BBB damage caused by hypoxia or vascular endothelial growth factor exposure, and reduce the expression of aquaporin-4 protein (AQP4) in vitro [92]. In the db/db mouse model of diabetes, metformin significantly decreased Aβ influx on BBB and neuronal apoptosis and increased intracerebral perfusion of Aβ as well as the expression of the low-density lipoprotein receptor-related protein 1 involved in Aβ efflux [93]. In rats’ traumatic brain injury (TBI) model, metformin inhibits TBI-mediated secondary injury through AMPK phosphorylation and improves BBB and neurobehavioral function [94]. Metformin significantly counteracts cigarette smoking-induced downregulation of tight junction protein and loss of BBB integrity by regulating Nrf2 expression [95]. In the cecal ligation and puncture (CLP) model of sepsis, metformin inhibits inflammation, increases tight junction protein expression, improves BBB function, and alleviates CLP-induced brain damage [96]. On the contrary, metformin can restore the cognitive function of mice fed with high fat and high fructose, but its protective effect on BBB is insignificant [97].

### 4.2. Transport of Metformin

Metformin is a biguanide derivative and exists as the hydrophilic cationic species at physiological pH values. Metformin is not bound to plasma proteins [98] and is excreted unchanged in the urine. The absorption, metabolism, distribution and renal excretions of metformin are mainly mediated by solute carrier transporters, a family of more than 300 membrane-bound proteins [99]. Among metformin transporters, organic cation transporters (OCTs) occur in the enterocyte, hepatocyte and renal tubule cells [99]. In addition to OCT, there are also multidrug and toxin compound extrusion-1 and -2 (MATE-1 and -2), plasma membrane monoamine transporter (PMAT), and thiamine transporter 2 (THTR-2) [100]. In humans, three OCT subtypes (OCT1, OCT2 and OCT3) have been isolated by cloning from diverse organs, including the brain. In the brain, OCT2 and OCT3 are mainly located in central neurons, and OCT3 is more widely distributed and also exists in astrocytes [101,102]. Furthermore, in previous studies, OCT1 and OCT2 are believed to exist in the BBB to help metformin enter the brain, while OCT2 and OCT3 regulate the concentration of metformin in the brain interstitium [103,104,105]; however, a recent study improved the separation and enrichment of cerebral microvessels, reducing the pollution of neurons and astrocytes by 31 and 7 times [106]. It was found that OCT1 and OCT2 were not expressed in mouse, rat or human cerebral microvessels [106]. In addition, functional studies conducted in the models of these three species further proved that there was no active OCTs vector in BBB [106]. Therefore, metformin does not seem to rely on OCT to achieve BBB penetration [106]. It is well established that metformin can rapidly cross BBB, but the transport mechanism of BBB osmosis is still controversial and needs further verification.

### 4.3. Metformin in Neuron

The CNS is undoubtedly the most elusive and delicate entity in our body. Neurons are the most important cell type in the CNS and have unique functions. Unlike skin, liver or muscle cells, neurons are highly differentiated cells in the CNS that cannot regenerate after disease, ischemia or brain injury. After decades of research, neuropathologists have found that in some neurological disorders, discrete groups of neurons are lost or damaged and characteristic protein aggregates in neurons, such as dopaminergic neuron loss in PD and amyloid plaques in AD [107,108]. The discovery of target neurons and pathological protein aggregation in these diseases provides a necessary theoretical basis for developing related drugs. As mentioned above, metformin has some effects on several neurological disorders, such as AD, PD, HD, MDD and FXS. 

Metformin has been reported to regulate the expression of abnormal proteins in the brain by autophagy. Autophagy is a lysosomal degradation process to recover obsolete cellular components and eliminate damaged organelles and protein aggregates. Neurons are more vulnerable to autophagy-related gene mutations because of their broad axon cytoplasm and lack of mitosis. Without effective autophagy, neurons accumulate ubiquitinated protein aggregates and eventually become degenerated [109,110]. Most neurodegenerative diseases are characterized by intracellular or extracellular aggregation of misfolded proteins, such as Aβ and tau in AD, α-synuclein in PD, huntingtin in HD and transactive response DNA-binding protein-43 (TDP-43) in amyotrophic lateral sclerosis (ALS) [111]. In the complex network of autophagy regulatory pathways, mTORC and AMPK signaling pathways serve as central nodes, integrating the metabolic signals and energy states of cells [112,113]. mTORC1 prevents autophagy mainly through suppressing phosphorylation of unc-51-like autophagy-activating kinase 1/2 (ULK1/2) and class III phosphatidylinositol 3-kinase (class III PtdIns3K complexes) [112]. The phosphorylation of its substrate RPS6KB1/S6K at Thr389 is a common marker for mTORC1 activity [112]. AMPK not only acts on the ULK1/2 and class III PtdIns3K complexes but also inhibits mTORC1 activity [114,115]. Phosphorylation on Thr172 of the AMPK catalytic subunit alpha and ACC on Ser79 are two common indicators of AMPK activity [113]. As mentioned above, metformin mainly influences neurodegenerative diseases such as AD. In a recent study, using double-transgenic APP/PS1 mice, metformin increased AMPK activity and decreased Aβ secretion, but did not increase the autophagic flux as rapamycin did [116]. It is worth noting that basal AMPK activity is necessary for normal autophagy activity [116]. These results suggest that metformin has a potentially complex regulatory mechanism that affects the production of abnormal proteins [116]. In a mouse model of tauopathy, chronic metformin treatment-induced PP2A expression through the AMPK/mTOR pathway reduced tau phosphorylation in the cortex and hippocampus of tau-P301S mice [117]; however, metformin also increased the number of insoluble tau species and the number of inclusions with β-sheet secondary structure in the cortex of P301S mice, and promoted the aggregation of recombinant tau protein in vitro [117]. Metformin also induces caspase 3 activation to enhance tau cleavage and damage synaptic structures [117]. In the APP/PS1 mouse, metformin effectively reduces brain Aβ plaque accumulation levels by stimulating transforming growth factor beta-activated kinase 1 (TAK1)—inhibitory kappa B kinase α/β (IKKα/β)—heat shock cognate protein 70 (Hsc70) signaling pathway to induce chaperone-mediated autophagy (CMA) activation, which is a lysosomal-dependent selective degradation pathway involved in the pathogenesis of cancer and neurodegenerative diseases [118]. In the db/db mouse model of diabetes, metformin inhibited the increase of total tau, phosphorylated tau and activated c-jun N-terminal kinase (a tau kinase), and mitigated the decrease of synaptophysin in the hippocampus [119]. In the SAMP8 mice of the AD model, metformin reduces the level of tau hyperphosphorylation possibly through inhibiting protein kinase R-like endoplasmic reticulum (ER) kinase (PERK) pathway, calpain 1, GSK3β and cyclin-dependent kinase 5 (Cdk5) activities [120]. 

In animal models of PD, metformin treatment substantially protects dopaminergic neurons from MPTP [121,122], rotenone [123], or 3, 4-methylenedioxymethamphetamine (MDMA) toxicity [124]. In PD, α-synuclein becomes insoluble and accumulates in the soma (Lewy bodies) and processes (Lewy neurites) of neurons to form intracellular inclusions in the wrong fold state. The key event in the formation of Lewy bodies is the phosphorylation of α-synuclein at Ser 129 [125]. Although metformin is a potent activator of AMPK, metformin significantly reduces the level of phospho-ser129 α-synuclein via mTOR-dependent PP2A activation [126]. PP2A is considered to be the primary α-synuclein phosphorylase [127]. Studies have reported that metformin enhances PP2A activity by increasing the C subunit’s methylation and reducing α-synuclein phosphorylation [128]. In addition, in the brains of PD patients, a study found that the ratio of methylation and demethylation of PP2A was significantly reduced, highlighting the important role of PP2A in α-synuclein hyperphosphorylation and aggregation [129]. Notably, Bayliss et al. found that the neuroprotective effect of metformin against MPTP neurotoxicity also occurred in AMPK knockout mice, supporting that AMPK activation was not associated with metformin neuroprotection [122]. In neurons with persistent prion infection, metformin significantly reduced cellular prion protein load and inhibited prion transforming activity, which may be explained by higher levels of autophagy [130]. Metformin improved propofol-induced HT-22 cell apoptosis and downregulated caveolin-1, a class of membrane proteins involved in the activation of autophagy [131]. Metformin reduced the abnormal HD protein load and fully restored the early network activity patterns characterized by increased activity, enhanced synchronicity, and hyperactive neurons [21].

Metformin may also play a neuroprotective role by improving mitochondrial homeostasis. RNAi-mediated knockdown of *Caenorhabditis elegans* (*C. elegans*) bcat-1 increases mitochondrial respiration and induces neuronal oxidative damage through an mTOR-independent mechanism, while metformin administration can correct abnormal mitochondrial respiration and reduce neurodegeneration of dopaminergic cell bodies and neurites [62]. In the Aβ-induced model of mitochondrial dysfunction in transgenic *C. elegans*, metformin reverses metabolic deficits associated with mitochondrial dysfunction and reduces protein aggregation [132]. In mice with insulin resistance induced by a HFD, metformin enhances insulin action by reversing the reduced ATP production and oxidative stress in an AMPK-independent way [133]. Metformin significantly improved H_2_O_2_-induced cell death by restoring abnormal intracellular reactive oxygen species (ROS), lactate dehydrogenase and mitochondrial membrane potential through activation of AMPK in neuronal PC12 cells and primary hippocampal neurons [134]. In a neuron-specific Ndufs3 conditional KO (cKO) mouse model, deletion of Ndufs3 in forebrain neurons reduced complex I activity, altered brain energy metabolism, and impaired motor performance [135]. Chronic metformin treatment did not significantly alter the metabolic status of AMPK and mTOR pathways and oxidative phosphorylation function in Ndufs3 cKO mice, but delayed the onset of neurological symptoms observed in Ndufs3 cKO mice [135]. Metformin reverses chemotherapeutic resistance by slightly inhibiting mitochondrial respiration [136] and is associated with tumor necrosis factor type 1 receptor-associated protein (TRAP1)-related pathways [137]. Metformin rescues mitochondrial phenotypic changes caused by TRAP1 loss, including recovery of mitochondrial membrane potential, mitochondrial nuclear protein imbalance, and mitochondrial unfolded protein response (mtUPR) upregulation [137]. Mitochondrial nuclear protein imbalance can activate stress signals through mtUPR and thus affect mitochondrial function and control longevity [138,139]. Metformin improves mitochondrial function through upregulation of chaperone protein and reduces carbonylation and oxidation of whole-brain proteins, which are markers of neuronal oxidative stress [140]. In summary, metformin plays a neuroprotective role by correcting mitochondrial respiration and improving oxidative stress through a variety of complex mechanisms.

### 4.4. Metformin in Astrocytes

Astrocytes account for about 30% of mammalian CNS cells and are the most abundant CNS cells. The functions of astrocytes mainly include regulating cerebral blood flow, maintaining neurotransmitter homeostasis, and regulating synaptic metabolism and neurotrophic support [141,142,143,144]. Reactive astrocytes undergo morphological, molecular, and functional remodeling in response to injury, disease, or infection of the CNS [145]. Metformin has been shown to inhibit reactive astrogliosis in many CNS injury models. In late middle aging mice, metformin treatment improved cognitive function, reduced hippocampal microglial activation and astrocyte hypertrophy, and reduced proinflammatory factor levels, along with AMPK activation and mTORC inhibition [146]. In hypoxia and glucose-deprived rats, metformin restricted cortical astrocyte apoptosis and increased cell viability, along with AMPK activation [147]. Metformin reduces ER stress and inflammation induced by high glucose in rat astrocytes by inhibiting caveolin1/AMPKα complex [148]. Metformin decreased the expression of AQP4 protein in cultured astrocytes, involved in AMPK activation and nuclear factor-κB (NF-κB) inhibition [92]. Taken together, inhibition of reactive astrogliosis by metformin is at least partially AMPK dependent. 

Reactive gliosis is an essential part of the neuroinflammatory process, which is also considered an important event in the pathogenesis of AD [149]. There is a close relationship between glial activation, proinflammatory factor release and neuronal injury. In human neural stem cells, metformin inhibited advanced glycosylation end product-induced inflammation and rescued the transcript and protein expression levels of ACC and IKK [150]. Metformin decreased glial activation induced by status epilepticus, downregulated mRNA levels of proinflammatory cytokines and chemokines, and improved BBB permeability and hippocampal neuron density, partly mediated by the mTOR pathway [151]. Metformin inhibits microglial activation in general, and immune reactivity of proinflammatory marker and anti-inflammatory marker [65]. In addition, metformin reduces the phosphorylated form of mitogen-activated protein kinases (pMAPKs) and ROS production by inhibiting nicotinamide adenine dinucleotide phosphate (NADPH) oxidase [65]. Metformin significantly reduced neuroinflammation and hippocampal neuron loss in diabetic animals, improving spatial memory [152]. Metformin downregulates the levels of apoptotic and proinflammatory factors, and reduces oxidative stress to protect the survival of striatum neurons after intracerebral hemorrhage [153]. Metformin significantly reduced neuroinflammation, reactive gliosis, and loss of hippocampal neurons in diabetic animals, resulting in improved spatial memory [152]. Metformin reduces brain damage in pneumococcal meningitis by reducing excessive neuroinflammatory responses and protects spiral ganglion neurons in the inner ear [154]. After permanent middle cerebral artery occlusion, chronic metformin preconditioning significantly reduced infarct volume, improved neurological deficits, reduced levels of inhibitory proinflammatory cytokines, and induced nitric oxide synthase in the periinfarct area [155]. In conclusion, metformin’s neuroprotective effects depend at least in part on inhibition of neuroinflammation and reactive gliosis.

In astrocytes treated with metformin, the rate of tricarboxylic acid (TCA) cycle and TCA cycle intermediates and derivatives were significantly reduced, and complex I-mediated mitochondrial respiration was impaired [156]. In diabetic rats after ischemic stroke, metformin therapy inhibited the reduction of sensorimotor deficits and prevented swelling and astrocyte protuberance around the infarct area [157]. In the astrocytoma model, metformin inhibited not only the NaN_3_-induced glycolysis, but also the migration of glycolytic cells [158]. Metformin can prevent oxaliplatin-induced intraepidermal fiber degeneration, gliosis and sensitivity changes in rats [159]. Metformin can also improve astrocyte and microglia proliferation in sporadic AD model rats [160]. Metformin reversed chronic corticosteroid-induced depression-like behavior changes and downregulation of glucocorticoid receptors in cultured rat prefrontal cortical astrocytes [161]. Metformin significantly increased the number of nuclear positive neurons in the CA1 region of ischemia/reperfusion rats, and decreased the number of glial fibrillary acidic protein-positive astrocytes [162]. One study showed that metformin enhanced astrocyte Ca^2+^ signaling and astroglia-driven regulation of synaptic plasticity [163]. In conclusion, metformin’s neuroprotective effect is closely associated with improved reactive astrogliosis. 

PEN2, recently identified as a metformin target, is thought to play an important role in AD pathology as an essential component of the γ-secretase complex that generates Aβ peptide [164]. In an oligodendrocyte-specific PEN2 cKO mouse model, loss of PEN-2 inhibits the Notch signaling pathway to upregulate signal transducers and transcriptional activators 3, thereby triggering the activation of GFAP and promoting differentiation of oligodendrocyte precursor cells to astrocytes [165]. In N2a cells overexpressing the mutant human APP gene, the downregulation of PEN2 decreased APP expression, while angiotensin-1 increased the secretion of Aβ42 through the FOXA2/PEN2/APP pathway [166]. 

### 4.5. Metformin in Synaptic Transmission

Synapses are the special structure between neurons, making communication between neurons possible. It is the fundamental component of neural network function. Neurotransmitters are released from the presynaptic axons into the synaptic cleft, binding to and activating receptors on postsynaptic membranes to transmit signals [167]. Activity-dependent regulation of the efficiency of synaptic transmission between neurons, often called synaptic plasticity, plays an essential role in brain development and function, primarily learning and memory [168]. Synaptic dysfunction has recently been recognized as the basis of some neurological disorders. Metformin, as a potential drug for treating some neurological disorders, is gradually recognized for its role in regulating synaptic transmission and plasticity.

In patients with MDD or bipolar disorder, abnormalities in the balance of excitation and inhibition (E/I balance) may contribute to abnormal functional connectivity patterns in brain networks. Neural network dysfunction is associated with altered levels of glutamate and gamma-aminobutyric acid (GABA) in the brain, and has been identified in studies of depression in animals and humans [169]. Metformin can promote the membrane insertion of GABA_A_ receptor and enhance the inhibitory synaptic neurotransmitter function and micro-inhibitory postsynaptic currents (mIPSCs) in cultured rat hippocampal neurons by activating AMPK-FOXO3A signaling pathway and increasing the expression of GABA_A_ receptor-associated protein [170]. In a rat model of diabetic epilepsy, metformin corrected the abnormal level of glutamate and GABA values in the hippocampus [171]. In an open-label study, increased corticospinal inhibition mediated by the GABA_A_ and GABA_B_ mechanisms was observed by transcranial magnetic stimulation in 15 FXS patients with metformin treatment, suggesting the potential of metformin in modifying GABA-mediated inhibition [172]. Glutamate excitatory toxicity in nutrient-deficient cells was mitigated after metformin treatment, mediated partly by downregulation of AMPK and subsequent reduction in autophagy [173]. Similarly, metformin directly inhibits glutamate-induced neuronal excitotoxicity by regulating autophagy and MAPK phosphorylation [174]. In the LPS-induced depression mouse model, metformin administration reduced presynaptic glutamate release and decreased the miniature excitatory postsynaptic currents (mEPSCs) frequency of hippocampal pyramidal neurons [80]. Metformin treatment restored excitatory synaptic activity in hippocampal sections to normal levels and rescued exaggerated metabolic glutamate receptor-dependent long-term depression (LTD) of synaptic transmission in *Fmr1* −/Y mice of FXS mouse models [85]. In one of our previous in vitro studies, we treated hippocampal slices with metformin and found that metformin treatment has no effect on GABAergic transmission onto CA1 pyramidal neurons [175]. Metformin treatment significantly increased the frequency, but not the amplitude, of mEPSCs, while the frequency and amplitude of mIPSCs were not changed [175]. Paired-pulse ratio analysis showed that presynaptic glutamate release was enhanced, but the excitability of CA1 pyramidal neurons was not changed [175]. In conclusion, metformin may affect glutamatergic and GABAergic synapses by directly regulating the number of neurotransmitters released and changing the expression level of receptors on the postsynaptic membrane.

In addition to these two important neurotransmitters, other neurotransmitters play an indispensable role in the central nervous system, such as acetylcholine (Ach), 5-HT and dopamine. When the local injection of metformin by retrodialysis, Ach has a short-term increase in the hypothalamus [90]; however, both doses of metformin did not increase acetylcholinesterase (AchE) activity in the hippocampus and cortex of mice; however, metformin normalized Ach cleavage in the hippocampus, and inhibited AchE activity in vitro [160]. Metformin can reverse the learning and memory impairment induced by scopolamine, but do not affect the inhibition of scopolamine-induced changes in Ach levels [176]. In *C. elegans* expressing human Aβ42, metformin can reduce the hypersensitivity to 5-HT induced by Aβ expression in neurons [177]. Metformin may have antidepressant effects by decreasing circulating branched-chain amino acids level and promoting serotonergic neurotransmission in the hippocampus [178]. In HFD-induced insulin-resistant mice, metformin stimulated 5-HT neurons excitability and 5-HT neurotransmission [81]. Metformin can induce the release of 5-HT in human duodenal mucosa biopsy specimens [179]. In some animal models of PD, metformin upregulated dopamine in the mouse brain [128,180]. In addition, metformin saved the MDMA-induced dopamine transporter decline [124]. In summary, metformin appears not only to improve synaptic transmission in pathological conditions broadly, but also to regulate E/I balance in neural networks. 

Besides, metformin also helps the morphological improvement of neurons under certain pathological conditions. In a rat model of metabolic syndrome (MS), the morphology of hippocampal neurons in MS rats was improved by metformin administration [181]. In detail, metformin restored the decline of dendritic length and dendritic spine density induced by a high-calorie diet in the hippocampus [181]. Metformin treatment prevented transcriptional changes in the medial prefrontal cortex and contributed to morphological changes in the neurite plasticity of CA1 pyramidal neurons [79]. Metformin significantly upregulated BDNF expression by increasing histone acetylation of the BDNF promoter, which is attributed to the activation of AMPK and CREB [79]. Metformin preconditioning partially restored sevoflurane exposure-induced significant reductions in hippocampal synaptic density and integrity through AMPK-ULK1-dependent autophagy in aged mice [182]. Metformin prevented the cisplatin-induced reduction in the number of dendritic spines and branches of neurons [183]. Metformin administration for 10 d corrected the dendritic abnormalities in *Fmr1*−/y mice of FXS model [85]. In the AlCl_3_-induced mouse neurodegeneration model, metformin normalized synaptic protein expression and significantly increased post-mitotic NeuN-positive neurons in the hippocampus [184]. In cultured primary cortical neurons, metformin treatment reduced postsynaptic density-95 (PSD-95), and significantly reduced the number of overlapping immunoreactive clusters of presynaptic synapsin I and postsynaptic PSD-95 proteins, suggesting an overall loss of synaptic buttons. In tau-P301S mice, synapsin I and PSD-95 levels did not change after chronic metformin treatment, but synaptophysin expression was significantly reduced [117]. Metformin ameliorates synaptic defects by suppressing Cdk5 hyperactivation in the hippocampus of APP/PS1 mice, and rescues various synaptic abnormalities, including spine loss, suppression of surface GluA1 trafficking and reduced basal synaptic transmission [185]. Thrombospondin-1 (TSP-1) is a protein secreted by astrocytes and a key factor regulating the development of dendritic spines and synaptogenesis [186]. In a clinical trial, metformin treatment can correct lower TSP-1 levels in patients with PCOS through the NF-κB and ERK1/2/ERK5 pathways [187]. In astrocytes exposed to ammonia, metformin increased synaptophysin levels and alleviated ammonia-induced reduction in intra- and extracellular levels of TSP-1 in astrocytes [188]. In conclusion, metformin alleviates synaptic morphological defects in various pathological conditions.

In HFD-fed prediabetic rats, metformin treatment restored the normalized field excitatory postsynaptic potential (fEPSP) slope and increment of the fEPSP slope of high-frequency stimulated long-term potentiation (LTP), suggesting the improved hippocampal synaptic plasticity by metformin [189]. Metformin also mitigated the effect of Aβ mediated-LTP in rats fed a high-fat diet, including significantly reduced population spike amplitude and EPSP slope [190]. Taken together, metformin not only resists the changes in synaptic morphology and synaptic number induced by external stressors, but also enhances synaptic plasticity.

## 5. Conclusions

**Figure 2:**
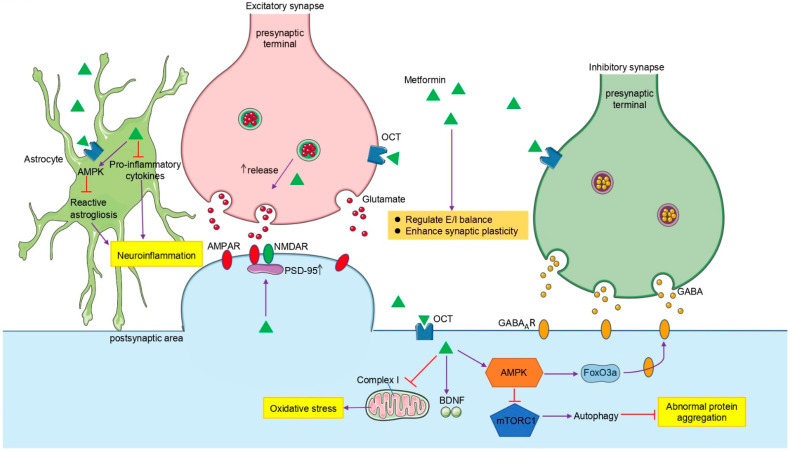
Potential mechanisms underlying the actions of metformin in the brain. In neurons, metformin regulates autophagy by AMPK/mTORC1 signaling pathway to alleviate abnormal protein aggregation. In addition, metformin decreases oxidative stress by regulating mitochondrial homeostasis. In astrocytes, metformin suppresses neuroinflammation by inhibiting reactive astrocyte proliferation and proinflammatory factors. In synaptic transmission, metformin may regulate E/I balance by directly regulating the amount of presynaptic neurotransmitter release or altering the expression level of receptors on the postsynaptic membrane. Metformin significantly ameliorates synaptic morphological defects and enhances synaptic plasticity in a variety of pathological conditions. AMPK, 5’-AMP-activated protein kinase; mTORC1, mechanistic target of rapamycin complex 1; AMPAR, AMPA-type glutamate receptor; NMDAR, NMDA receptors; PSD-95, postsynaptic density protein 95; BDNF, brain-derived neurotrophic factor; FoxO3a, forkhead box O3a; GABA, gamma-aminobutyric acid; GABA_A_R, GABA type A receptor.

In conclusion, we summarized the clinical application and efficacy of metformin in various neurological disorders, and analyzed the effect of mainstream animal model experiments (Table 1). It has been found that metformin has a broad neuroprotective effect, but further validation in some animal models and exploration of its underlying mechanisms are needed. We focused on metformin’s role in neuron, astrocyte and synaptic transmission. Metformin can improve synaptic transmission, affecting neural circuits and regulating E/I balance in neural networks (Figure 2). Finally, further studies are needed to investigate the exact mechanism of metformin’s neuroprotective effects and the heterogeneous sources of side effects. Metformin may be an attractive drug for preventing neurological disorders in the future because of its few clinical side effects.

## Figures and Tables

**Table 1:** Effects of metformin on neurological disorders.

Neurological Disorders	Clinical Trials	Animal or Cellular Studies		
		Model	Effects	Potential Mechanisms
Alzheimer’s disease (AD)	In a double-blinded, placebo-controlled crossover pilot study, non-diabetic subjects with AD showed improvement in executive function after taking metformin for 8 weeks, with trends indicating improved learning/memory and attention [48]. Oral metformin (mean average dosage of 500 mg per day) reduced the risk of developing AD in T2DM patients to 0.76 [40]. In patients with T2DM, metformin users performed better on immediate and delayed memory over time [52]. In a meta-analysis, the incidence of cognitive impairment was significantly reduced in metformin diabetic patients (Odds ratio = 0.55, 95%CI 0.38–0.78), and dementia was also significantly reduced (Hazard ratio = 0.76, 95%CI 0.39–0.88) [191]. Among diabetic patients, metformin users have a lower risk of developing AD than other hypoglycemic drug users [41,192]. Conversely, long-term use of metformin has been associated with a higher risk of AD in some studies [49,50,193].	APP/PS1 mice	Metformin (200 mg/kg, i.p. for 14 days) attenuated spatial memory deficit, neuronal loss, increased Aβ plaque and chronic inflammation [53].	Metformin activates AMPK/mTOR/S6K/BACE1 and AMPK/P65 NF-κB.
			Metformin (drinking water containing metformin for 12 weeks) effectively reduces accumulated Aβ plaque levels and reverses the molecular and behavioral phenotypes of AD [118].	Metformin activates chaperone-mediated autophagy by TAK1-IKKα/β-Hsc70-CMA.
			Metformin (200 mg/kg/day, oral administration for 8 weeks) improve learning and memory ability, neurological dysfunction and oxidative stress, and reduced Aβ levels and increased the expression of synaptic-related genes [194].	Metformin activates AMPK signaling pathway and upregulates the insulin-degrading enzyme.
			Metformin treatment (200 mg/kg, i.p. for 10 days) restoring spinal density, surface GluA1 transport, LTP expression, and spatial memory [185].	Metformin inhibits cyclin-dependent kinase 5 hyperactivation by inhibiting Calpain, leading to inhibition of tau hyperphosphorylation.
		APP/PS1 mice injected with tau aggregates	Metformin (drinking water containing metformin for 2 months) reduced Aβ load and tau pathological changes and increased the number of microglia around Aβ plaques [195].	Metformin improves Aβ pathology and limits tau transmission by enhancing autophagy.
		SAMP8 mice	Metformin (20 mg/kg/sc or 200 mg/kg/sc, i.p. for 8 weeks) improved memory of spontaneous onset AD by decreasing APPc99 and p-tau at both concentrations [54].	Metformin may reduce tau phosphorylation by regulating the protein kinase C and GSK3β.
		Primary cortical neurons from wild-type and human tau transgenic mice	Metformin (2.5 mM) induces PP2A activity and decreases tau phosphorylation at PP2A-dependent epitopes in vitro and in vivo [55].	Metformin induces tau dephosphorylation through direct activation of PP2A, and this pathway is independent of AMPK activation.
		Primary cortical neurons and N2a cells	Metformin (1~10 μM) increased the production and secretion of Aβ by upregulating BACE1 promoter activity [56].	Metformin affects Aβ levels and BACE1 transcription in an AMPK—dependent manner.
Parkinson’s disease (PD)	Compared with untreated diabetic patients, there is no difference (HR 0.95) in PD risk when metformin is used alone, but sulfonylurea-alone increases the risk (HR 1.57), while the combination of the two can reduce the risk (HR 0.78) [18]. In patients with T2DM, metformin users were at higher risk of PD (HR: 2.27, 95% CI 1.68–3.07) [7]. Compared with metformin alone, glitazone was associated with a significantly lower incidence of PD (HR 0.72; 95%CI 0.55–0.94) [19].	MPTP-induced PD mice	Long-term metformin treatment (500 mg/kg, oral administration for 21 days) significantly ameliorates MPTP-induced motor injury and dopaminergic neuron death [61].	Metformin improved oxidative stress and upregulated BDNF levels.
		6-OHDA-lesioned mouse model of PD	Metformin (100 and 200 mg/kg, oral administration for 10 days) co-treatment with L-DOPA suppresses the development of dyskinesia [63].	Metformin induced enhancement of mTORC, dopamine D1 receptor and ERK1/2 signaling, and normalized the Ak/GSK3β signaling.
			Metformin (100 mg/kg and 200 mg/kg, oral administration for 4 weeks) treatment can effectively improve the motor symptoms of PD mice [64].	Metformin induces the activation of AMPK and BDNF signaling, and regulates the astrocyte activation.
		Bcat-1 knockdown worm model of PD	Metformin (50 μM) treatment could correct the abnormal mitochondrial respiration and evidently rescued dopamine neuron viability [62].	Metformin can activate AMPK and upregulate BDNF, and inhibit reactive astrocytes.
		LPS-induced rat model of PD	Metformin (150 mg/kg, oral administration for 7 days) generally inhibited the activation of microglia and the expression of inflammatory cytokines [65].	Metformin reduces mitochondrial respiration through the mTORC-independent mechanism
		Haloperidol-induced catalepsy model of PD	Metformin (20~100mg/kg, oral administration for 21 days) significantly attenuated memory deficit, oxidative stress and lipid peroxidation [66].	Metformin inhibits the pMAPKs and ROS production by inhibiting NADPH oxidase
Huntington’s disease (HD)	HD patients with T2DM receiving metformin had better cognitive test results than those without diabetes not taking metformin [25].	Hdh150 knock-in mouse model of HD	Metformin (drinking water containing metformin 5mg/mL for 16–24 days) can reduce the aberrant huntingtin load and completely restore the early network activity pattern and abnormal behavior [21].	Metformin at low doses did not activate AMPK, but instead activated the mTOR/PP2A pathway
		zQ175 mouse model of Huntington’s disease	Metformin (drinking water containing metformin 2mg/mL for 3 months) improved motor. upregulated the expression level of BDNF, and reduced reactive astrocytes and microglia [69].	Metformin treatment reduces pERK1/2 expression
		Worm models of polyglutamine toxicity	Metformin (2 mM) prevents aggregation of abnormal aberrant huntingtin and neuronal impairment [70].	Metformin improves neuronal toxicity in an AMPK- and lysosome-dependent mechanism
		HEKT cells overexpressing huntingtin	Metformin (1 mM or 2.5 mM) reduces mutant huntingtin translation rate and S6 phosphorylation [21].	Metformin regulates huntingtin by mTOR/PP2A pathway
Major depressive disorder (MDD)	In a large-scale study of adolescents with severe mental illness, metformin add-on was associated with significantly fewer aggressive and impulsive problems [22]. Metformin has been associated with a lower incidence rate of depression and improve symptoms of depression in several other clinical studies [72,73,74,75,76,77,78].	LPS-induced mice model of MDD	Metformin (200 mg/kg, i.p. for 10 days) administration ameliorated depressive-like behaviors [80].	Metformin reduces increased mEPSC frequency and presynaptic glutamate release.
		HFD-induced insulin-resistant mice	Metformin (drinking water containing metformin 300 mg/kg/day for 7 weeks) alleviates HFD-induced anxiety-/depressive-like behaviors [81].	Metformin promotes 5-HT neurotransmission by reducing circulating branched-chain amino acids.
		CSDS mouse model of MDD	Metformin (200 mg/kg/day, oral administration for 21 days) alone relieved depression-like behaviors and improved CSDS-induced synaptic defects in mice [79].	Metformin upregulates BDNF expression by activating AMPK/CREB signaling.
Fragile X syndrome (FXS)	In seven FXS patients, metformin treatment was associated with improvement in irritability, social reactivity, hyperactivity, and social avoidance [83].	*Fmr1*-KO mouse model of FXS	Metformin reverses the social behavior defects, rescues long-term depression and impaired spine morphology [85].	Metformin selectively normalizes ERK signaling, and the expression of MMP-9.
